# Anesthetic experience of an adult male with citrullinemia type II: a case report

**DOI:** 10.1186/s12871-016-0253-7

**Published:** 2016-10-11

**Authors:** Jung Ju Choi, Hong Soon Kim, Kyung Cheon Lee, Youseok Shin, Youn Yi Jo

**Affiliations:** Department of Anesthesiology and Pain Medicine, Gachon University Gil Medical Center, 1198 Guwol-dong, Namdong-gu, Incheon, 405-760 South Korea

**Keywords:** Adult onset citrullinemia type II, Hyperammonemia, Anesthesia

## Abstract

**Background:**

Citrullinemia type II is an autosomal recessive urea cycle disorder and a subtype of citrin deficiency. However, the management of recurrent hyperammonemia with neurologic symptoms in patients with citrullinemia type II is quite different from the management of other types of urea cycle disorders. In pats with citrullinemia type II, regional anesthesia might be a good choice for the early detection of hyperammonemic symptoms and addressing psychic stress.

**Case presentation:**

A 48-year-old male with adult onset citrullinemia type II was scheduled for urethral scrotal fistula repair. During the first operation, spinal anesthesia with conscious sedation using dexmedetomidine was used, a second operation was performed after confirmation of infection control and a stable neurologic condition. In this patient, dietary planning with close monitoring of serum ammonia level and close observation of neurologic conditions might lead to successful perioperative care.

**Conclusion:**

For anesthesia of patients with adult onset citrullinemia type II, close monitoring of neurologic signs and serum ammonia are important to reduce neurologic complications induced by hyperammonemia. Regional anesthesia with a proper dietary plan might reduce patient stress and prevent metabolic tragedy.

## Background

Citrullinemia type II is a subtype of citrin deficiency, and is an autosomal recessive urea cycle disorder characterized by recurrent uncompensated hyperammonemia with neurologic symptoms. Although the majority of urea cycle disorders are diagnosed during the neonatal period, citrullinemia type II manifests in children and adults [1]. This rare disease is related to the SLC25A13 pathogenic variant, and causes a liver-specific argininosuccinate synthetase deficiency [2]. Although serum ammonia levels are not consistent with clinical symptoms, hyperammonemia can lead to cerebral edema and neurologic injury [3], and thus, perioperative monitoring of serum ammonia levels and aggressive management to reduce serum ammonia levels are important.

Hyperammonemic crisis in patients with urea cyclic disorder can be provoked by infection, fever, preoperative fasting, surgery, or general anesthesia, which are commonly encountered during the perioperative period [4]. Moreover, the management of hyperammonemic encephalopathy in patients with citrullinemia type II differs substantially from what is required to treat other classic urea cycle disorders. In particular, the low protein diets and high amounts of carbohydrate normally provided as emergency regimens to patients with other urea cycle disorder are associated with risks in citrullinemia type II patients [5, 6], and thus, strict dietary planning is an important aspect of perioperative management.

No previous report has been issued in the English literature on anesthetic experience of an adult patient with citrullinemia type II. Here, we describe our experience and perioperative care of a patient with recurrent hyperammonemic encephalopathy related to citrullinemia type II.

## Case presentation

A 48-year-old male (178 cm, 60 kg, body mass index 18.9 kg/m^2^) was scheduled for urethral scrotal fistula repair. He had been diagnosed with anxiety disorder, imprisoned for illegal drug use, and diagnosed some 15 years previously with hepatitis C. Ten years before visiting our hospital, he experienced a mental status change, was hyperammonemic, but with normal liver function, and was diagnosed with citrullinemia type II based on biochemical assay results. He had never experienced ascites or esophageal varix, but had experienced recurrent uncompensated hyperammonemia, for which he was taking arginine and sodium benzoate. Prior to transfer to our hospital, he had been living in a sanatorium in a bed ridden state with slurred speech and motor weakness. Subsequent brain computed tomography demonstrated progressive diffuse brain atropy.

**Fig. 1:**
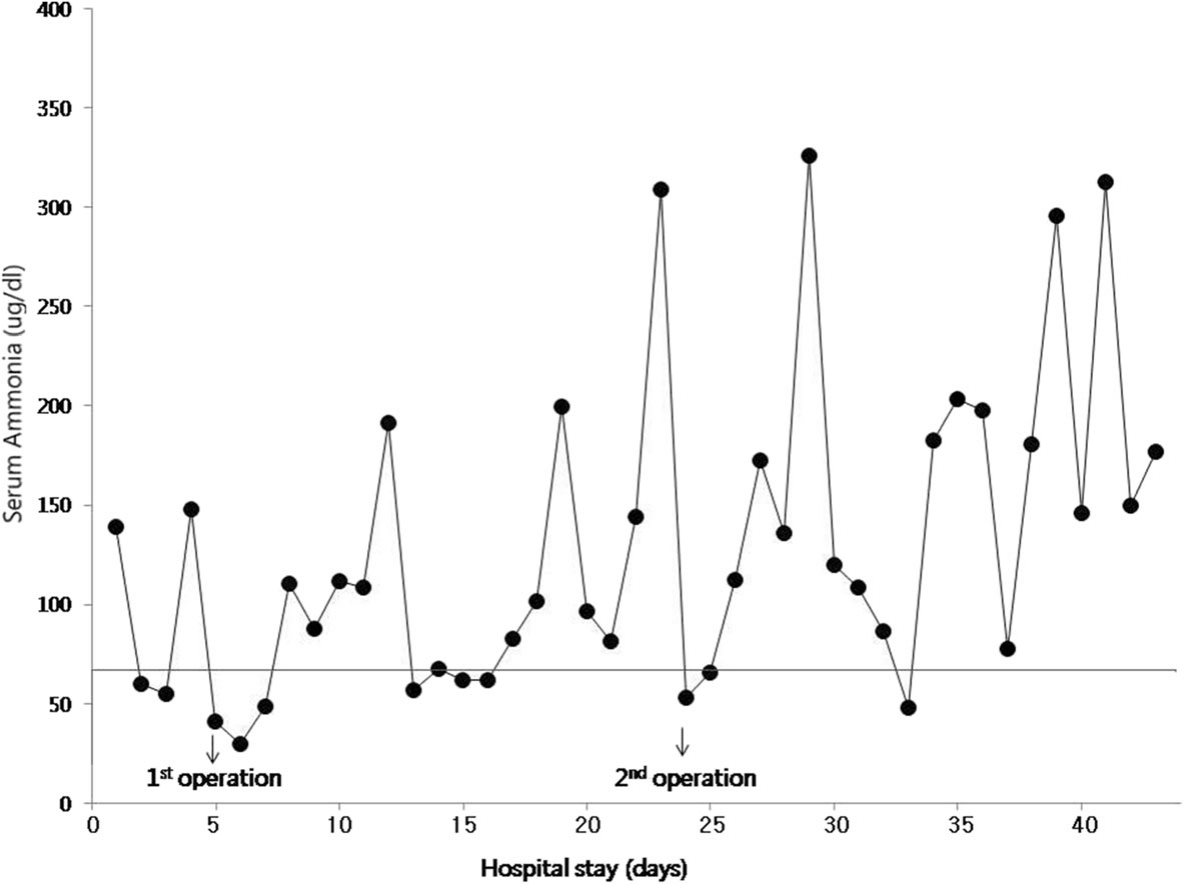
Perioperative serum ammonia level changes

The patient was admitted to our urology department for urethral fistula repair and cystolitholapaxy. Preoperative electrocardiographic and chest x-ray findings were normal, and pre- and post-operative serum ammonia levels were illustrated in Fig. 1 (normal value 12-66 μg/dL). Serum ammonia level fluctuations were controlled by daily lactulose enema and intravenous arginine and sodium benzoate. The medical team involved prescribed a 1000 kcal/day diet with a PFC ratio of 14 %:27 %:59 %. Preoperatively, his blood pressure was 100/50 mm Hg, heart rate 88 beats/min, and body temperature 39.3 °C, and he had a scrotal abscess. His preoperative laboratory data were as follows: hematocrit 31.3 %, platelet count 161 × 10^3^/ml, blood urea nitrogen (BUN) 10.4 mg/dl, creatinine (Cr) 0.5 mg/dl, prothrombin time (PT) 14.5 sec (INR 1.34), activated partial thromboplastin time (aPTT) 38.5 sec, serum albumin 2.4 g/dl, aspartate aminotransferase 88 IU/L, alanine aminotransferase 43 IU/L, total bilirubin 0.5 mg/dl, direct bilirubin 0.32 mg/dl, and serum gamma-glutamyl transferase 349 IU/L, the latter of which was moderately elevated (normal 0-72U/L).

Surgery was performed 5 days after admission. He was not administered premedication and in the operating theatre his vital signs were monitored by pulse-oximetry, ECG, and a non-invasive blood pressure device. Spinal anesthesia was performed using 12 mg of 0.5 % hyperbaric bupivacaine using a 25-gauge Quinke needle to block the T6 thoracic level. Dexmedetomidine was administered for sedation during surgery. Plasma solution (600 ml; sodium chloride 5.26 g, sodium gluconate 5.02 g, sodium acetate hydrate 3.68 g, potassium chloride 0.37 g, and magnesium chloride 0.3 g per liter; Plasma solution A inj., CJ Health Care, Korea) was infused during the 120 min operation, which involved open cystolitholapaxy, fistula repair, and removal of the abscess pocket, and estimated blood loss was 100 ml. No adverse event occurred during surgery. The surgical team planned a second-look operation after fever control had been achieved and confirmed the absence of bacterial growth in the surgical wound. No neurologic events of significance occurred before the second operation despite poorly controlled serum ammonia levels.

Twenty days after the first operation, while still hospitalized, the patient underwent a second operation under spinal anesthesia (T8 with 12 mg of 0.5 % hyperbaric bupivacaine) for wound repair and cystoscopic examination; 0.9 % normal saline was used as distension medium during the cystoscopic examination. During the 60 min operation, 400 ml of plasma solution was infused. The patient did not experience any change in consciousness level, despite fluctuating serum ammonia levels, during his 43-day hospital stay.

## Conclusion

Citrullinemia type II is an inherited metabolic disease that has been reported to have an incidence of 1 per 100,000 of the population in Japan and to be rare in America and Europe [1]. Under stressful medical or surgical conditions hyperammonemic symptoms can develop even in patients with no previous diagnosis of citrullinemia type II [7].

This condition is caused by a mutation in the SLC25A13 gene, which encodes citrin transporter, that can interfere with glycolysis, the urea cycle, gluconeogenesis, and galactose metabolism [1]. The clinical features of citrullinemia type II are hyperammonemia, neuropsychiatric symptoms, and hepatic dysfunction. Furthermore, more than 90 % of citrullinemia type II patients have a body mass index of < 20 kg/m^2^ [2, 8]. Unlike other urea cycle disorders, which are diagnosed during the neonatal period, citrullinemia type II occurs suddenly between the ages of 11 and 79 years [1]. Our patient had typical signs and symptoms of citrullinemia type II, that is, a history of anxiety disorder, neurologic symptoms, such as, motor dysfunction and slurred speech, a body mass index of 18.9 kg/m^2^, a diagnosis in adulthood, and recurrent hyperammonemic episodes in addition to inactive hepatitis C.

Alcohol intake or) stressful medical or surgical conditions can trigger hyperammonemic crisis in citrullinemia type II patients, and this can lead to life threatening events, such as, brain injury or even death [1, 7]. However, to the best of our knowledge, no report has been issued on perioperative concerns regarding patients with adult onset citrullinemia type II.

In our patient, a perioperative hyperammonemic event could have mimicked delayed emergence from general anesthesia [9, 10], Also, hypotonic, nonconductive, low-viscosity fluids like glycin might improve visualization during cystoscopy but present the risk of hyperammonemia, electrolyte disturbances, such as, extreme hyponatremia or brain edema. Furthermore, the absorption of distension media can cause headache, anxiety, confusion, seizures, and death, which are also the symptoms of hyperammonemic crisis [11, 12]. In fact, the early detection of extreme absorption of distension media or a hyperammonemic event is critical for early treatment. We selected spinal anesthesia to enable changes in mentality to be checked during surgeries and dexmedetomidine as a sedative, because of its suitability for conscious sedation [13]. Furthermore, normal saline was used as a distension media during cystoscopy.

Mots urea cycle disorders require low protein, high carbohydrate diets to reduce ammonia production, but citrullinemia type II requires a quite different diet plan. Because citrullinemia type II is caused by defective aspartate export from mitochondria to cytosol, the cytosolic nicotinamide adenine dinucleotide to oxidized nicotinamide adenine dinucleotide ratio (NAHD/NAD^+^) can increase abnormally, inhibit glycolysis, and caused difficulties associated with the metabolism of alcohol [2, 14], which is why patients with citrullinemia type II prefer diets rich in aspartate and asparagine, such as, beans and peanuts. High carbohydrate intake increases NADH/NAD^+^ ratio and disturbs ureagenesis by restricting aspartate supply to the urea cycle, resulting in severe hyperammonemia, and thus, PFC ratios of greater than 60 % should be avoided in citrullinemia type II patients [14]. For our patient, we recommended a PFC ratio of 14 %:27 %:59 %, restricted calories (in view of his bed-ridden state), and no glucose solution infusion.

Summarizing, for the anesthesia of patients with adult onset citrullinemia type II, changes in neurologic signs and the monitoring of serum ammonia are important for reducing hyperammonemia-induced neurologic complications. Given a well thought out plan for reducing stressful surgical conditions, dietary care should not be overlooked. Furthermore, regional anesthesia with proper sedation is probably be a good choice for the early detection of hyperammonemic symptoms and for the differential diagnosis of excess absorption of distention media in citrullinemia type II patients undergoing an urologic procedure.

## Abbreviations

aPTT: activated partial thromboplastin time
BUN: ratio blood urea nitrogen
Cr: creatinine
INR: international normalized ratio
NAHD/NAD^+^: nicotinamide adenine dinucleotide to oxidized nicotinamide adenine dinucleotide ratio
PFC ratio: Protein, fat, carb ratio
PT: prothrombin time
SLC25A13: Solute Carrier Family 25 Member 13
